# Integrative Analysis of miRNAs Involved in Fat Deposition in Different Pig Breeds

**DOI:** 10.3390/genes14010094

**Published:** 2022-12-28

**Authors:** Xiuxiu Zhang, Wanlong Huang, Yuntao Guo, Xiangyang Miao

**Affiliations:** State Key Laboratory of Animal Nutrition, Institute of Animal Sciences, Chinese Academy of Agricultural Sciences, Beijing 100193, China

**Keywords:** pig, RNA-seq, miRNAs, adipose tissue, PPAR

## Abstract

Background: miRNAs are a set of small, noncoding RNAs that bind to partially complementary sequences on target mRNAs. This leads to the post-transcriptional regulation of gene expression. Many studies have shown that microRNAs play critical roles in adipose cell differentiation and fat metabolism. The aim of this study was to explore the regulatory functions of miRNAs in fat deposition for the prevention and therapy of lipid metabolism-related diseases. Methods: The significant differences in the fat deposition of Laiwu (LW) pigs and Large White (LY) pigs were studied. To investigate the genetic relationships of miRNAs that regulate fat deposition, we performed a genome-wide analysis of miRNAs derived from subcutaneous adipose tissue of LW and LY pigs using RNA-seq. Results: There were 39 known miRNAs and 56 novel miRNAs significantly differential expressed between the two breeds of pigs. In the analysis of the Gene Ontology and KEGG pathways, predicted targets of these differentially expressed miRNAs were involved in several fat-associated pathways, such as the peroxisome proliferator-activated receptor (PPAR), mitogen-activated protein kinases (MAPK) and Wnt signaling pathways. In addition, ssc-miR-133a-3p, ssc-miR-486 and ssc-miR-1 each had a great impact on the development of porcine subcutaneous fat through the PPAR signaling pathway. Conclusions: We explored the role of differentially expressed miRNAs and studied the mechanisms of adipogenesis and fat deposition between two different pig breeds. In addition, these results also contribute to research relevant to human obesity.

## 1. Introduction

Obesity is a heterogeneous disease that is associated with comorbidities, such as type 2 diabetes mellitus (T2DM), cardiovascular disease and cancer [1]. Obesity has always been a major risk for human health. Therefore, it is urgent to study the mechanism of obesity and to develop effective treatment. In general, adipose tissue (AT) is a passive reservoir for energy storage that also functions as a mechanical and heat insulator and participates in the regulation of thermogenesis. However, AT synthesizes and secretes a variety of bioactive molecules, known as “adipokines”. They are involved in the regulation of glucose and lipid metabolism, energy homeostasis, feeding behavior, insulin sensitivity, inflammation, immunity, adipogenesis and vascular function or coagulation [2]. Many researches have studied the roles of dysfunctional adipose tissue in the pathogenesis of obesity. Therefore, it is necessary to study the function of adipose tissue to reveal the mechanism of obesity. 

The function of adipose tissue is closely associated with fat metabolism and adipocyte differentiation. Existing studies have shown that adipose cell differentiation and fat metabolism were strictly regulated by several transcription factors, including sterol regulatory element binding proteins, CCAAT/enhancer binding proteins and peroxisome proliferator-activated receptor γ. Some studies have shown that microRNAs (miRNAs) can play key roles in adipocyte differentiation and fat metabolism.

miRNAs are small non-coding RNAs (18–22 nt) that bind to partially complementary sequences on target mRNAs, thereby performing the post-transcriptional regulation of gene expression [3]. They play key roles in cell differentiation, apoptosis, metabolism and other important biological processes [4,5,6,7]. These include their effects on adipocyte differentiation and fat deposition. For example, a previous study [6] found that mir-14 is a dose-dependent regulator of Dystroglycan 1 (DAG) and Tumor Associated Gene (TAG) metabolism in Drosophila. Esau et al. reported that miR-143 is involved in adipocyte differentiation [8]. In addition, bta-miR-378 promotes the differentiation of bovine preadipocytes [9], ssc-miR-7134-3p regulates fat accumulation in castrated male pigs [10] and miR-429 inhibits porcine preadipocyte differentiation [11]. We identified the miRNAs which affect the adipocyte differentiation and fat metabolism in pigs.

Pigs are an excellent model for studying obesity and obesity-related diseases. The reason for this is the ability of pigs to create fat deposits in a manner similar to that of humans. In addition, the development, morphology and function of the normal cardiovascular system in swine are very similar to those in humans [12]. Like humans, pigs are omnivores, and develop spontaneous atherosclerosis with increased age. In addition, their lipoprotein profiles and metabolisms are similar to that of humans [13]. The Laiwu pig is a typical obese pig that easily deposits fat. It is a unique breed in the Shandong Province of China, with the characteristics of high adaptability, good meat quality, strong fecundity and good overall performance. Large White pigs, called Yorkshire pigs, originate from the United Kingdom, and have the characteristics of large size, high fecundity and adaptability, high feed conversion rate and dressing percentage. Large White pigs are one of the world-class lean meat-type pigs. These two breeds show significant differences in fat accumulation, and serve as ideal animal models for studying fat deposition and obesity.

Next-generation sequencing has been used to examine the transcriptome in order to identify differential expressions and opportunities to discover novel transcripts, including novel alternative isoforms and miRNAs [14,15,16,17,18,19,20,21,22,23,24,25,26,27]. Therefore, we compared miRNA profiles of the adipose tissue of the Laiwu pigs and Large White pigs using RNA-seq. The data were further analyzed to identify the miRNAs that were related to adipopexis. This study provides information on miRNAs that are involved in the fat accumulation in pigs, as well as their regulated target genes and pathways. This provides clues for solving obesity-related problems in humans. 

## 2. Materials and Methods

### 2.1. Ethics Statement

All procedures involving animals were approved by the animal care and use committee at the Institute of Animal Sciences, Chinese Academy of Agricultural Sciences, where the experiment was conducted. All of the experiments were performed in accordance with the guidelines and regulations formulated by the Ministry of Agriculture and Rural Affairs of the People’s Republic of China.

### 2.2. Sample Preparation

All animals were kept in Daqian Husbandry Co., Ltd., Laiwu City, China. Two breeds were analyzed: Laiwu pigs and Large White pigs. They were housed under the same conditions, including free access to water and food under natural lighting. Five healthy female pigs from each breed (150 days old) that were fed according to current nutritional needs (National Research Council, NRC, 1998) were selected. They were all healthy and in good physical condition, with the same weight within the breeds at slaughter age. The mean weights of the Laiwu pigs and Large White pigs were 35 kg and 90 kg, respectively. We collected subcutaneous adipose tissue after slaughtering the animals. Then, these tissues were frozen in liquid nitrogen. They were immediately stored at −80 °C for further RNA extraction. All of the experimental procedures were carried out under the authorization of the Chinese Ministry and Rural Affairs of the People’s Republic of China.

### 2.3. Library Preparation and miRNA Sequencing

Total RNA was extracted from subcutaneous adipose tissues of the two breeds using TRIzol (Invitrogen), as described by the manufacturer. We assessed the quality and quantity of RNA samples on a Bioanalyzer 2100 system through an RNA 6000 Nano kit (Agilent Technologies, Palo Alto, CA, USA) Qualified total RNA was stored at −80 °C for later miRNA separation. We constructed two small RNA libraries representing the two breeds (from a pool of 5 Laiwu pigs and a pool of 5 Large White pigs) based on the Illumina^®^ TruSeq™ Small RNA Sample Preparation protocol. We used 2 μg of total RNA from each sample to construct cDNA libraries. Small RNAs were isolated from the total RNA by denaturing polyacrylamide gel electrophoresis. The 5′-adaptor and 3′-adaptor were ligated to these small RNAs, followed by reverse transcription with SuperScript II Reverse Transcriptase (Invitrogen). Then, the cDNA was amplified by PCR, and purified to generate two small RNA libraries. We performed quality control analysis on the library using the Qubit™ dsDNA HS kit and a Qubit^®^ 2.0 Fluorometer. Then, we determined the library size and purity through the High Sensitivity DNA Chip and Agilent 2100 system. Finally, we sequenced qualified small RNA libraries through the single-read multiplex program on the Illumina HiSeq 2500 platform (Illumina, CA, USA) of the Beijing Biotechnology Corporation (Beijing, China). The two cDNA libraries from the two species (from a pool of 5 Laiwu pigs and a pool of 5 Large White) were named L and D, respectively.

### 2.4. Mapping and Annotation

Reference genome and gene model annotation files were directly downloaded from the genome website. To analyze the miRNA sequencing results, we initially filtered out the adaptor sequences and low-quality sequences from the raw reads using the fastx_toolkit (http://hannonlab.cshl.edu/fastx_toolkit/, accessed on 30 May 2022). We first aligned clean reads to the pig genome (NCBI, Sscrofa10.2) using the bowtie program (version 1.0.0) [28], and then searched against the Rfam database (http://rfam.xfam.org/, accessed on 30 May 2022) to remove rRNA, tRNA and snRNA. To identify the conserved miRNAs, we compared the remaining sequences with the mature miRNAs of humans and animals, including cows, sheep and pigs, in miRBase (Release 21; http://www.mirbase.org/, accessed on 30 May 2022). If there was a minimum 16-nucleotide match between the sequence read and the miRNA from the database, hits were considered real matches. The read sequences that matched with the reference mature miRNAs were annotated as miRNA candidates. Then, the miRNA candidates were clustered into categories based on sequence similarity. The sequences varying only in length and/or a few end nucleotides were grouped under the same miRNA identifier or related keywords. This is typically used to identify miRNAs (https://www.mdc-berlin.de/36105849/en/research/research_teams/systems_biology_of_gene_regulatory_elements/projects/miRDeep/documentation, accessed on 30 May 2022).

### 2.5. Identification of Novel miRNAs

We extracted 150 nucleotides for a sequence, flanking each side of these miRNA candidate sequences, to identify potential novel miRNAs. Secondary structures were predicted through the MIREAP program (http://sourceforge.net/projects/mireap/, accessed on 30 May 2022). If a hairpin structure with a free energy of hybridization lower than −20 kcal/mol was predicted, the RNA sequence was subjected to the miReap analysis. This predicted whether the input RNA sequence as a genuine pre-miRNA-like hairpin sequence. Any sequence that met the following three criteria was harvested: (1) mature miRNAs reside in one arm of the hairpin precursor, which are short of large internal loops or bulges; (2) the stem–loop structure is steady, and the free energy hybridization is lower than −20 kcal/mol; and (3) hairpins are located in intergenic regions or introns are considered as potential new miRNAs [28].

### 2.6. Differential Expression Analysis

To obtain the differentially expressed miRNAs, we used the RPM method to judge the expression level of the two samples. The DEGSeq R package (1.10.1) [29] was used to perform the differential expression analysis of miRNAs. This can provide statistical routines to determine the differential expression in the digital gene expression data through the model based on the binomial distribution. To control the false discovery rate, we corrected *p*-values through Benjamini and Hochberg’s approach. Differentially expressed miRNAs with a corrected *p*-value < 0.05 and a difference greater than 2-fold were selected for further analysis.

### 2.7. Target Prediction of Differentially Expressed miRNAs

In mammals, miRNAs pair imperfectly, complementary to the 3′-UTR region of the target genes, to repress the transcription of key regulators related to growth, differentiation and other important pathways. The second base to the eighth base of the miRNAs are generally considered as the ‘seed’ sequence. This is important for recognizing and binding the 3′-UTR regions of their targets. Therefore, to explore the potential function of the miRNAs that have significantly differential expressions in porcine subcutaneous adipose tissue, we applied the miRanda program (http://www.microrna.org/microrna/getDownloads.do, accessed on 30 May 2022) to predict their target mRNAs in the linux system based on the complementary pairing of bases. Based on the gene annotation information, the 3′-UTR sequences were extracted. The parameters were set as follows: score threshold at 140; energy threshold at −19 kcal/mol; scaling parameter at 4 and gap-open penalty at −9. A set of predicted target genes was designed for further analysis.

### 2.8. GO and KEGG Pathway Analyses of the miRNA Target Genes

Target genes of differentially expressed miRNAs were subjected to GO enrichment analysis by DAVID software (https://david.ncifcrf.gov/, accessed on 30 May 2022). The GO terms with corrected *p*-values less than 0.05 were significantly enriched terms, with all the porcine genes as the list of the background genes. KEGG is a database for understanding high-level functions and utilities of the biological system, such as the cell, the organism and the ecosystem, from molecular-level information, especially for large-scale molecular datasets generated by genome sequencing and other high-throughput experimental technologies (http://www.genome.jp/kegg/, accessed on 30 May 2022). The statistical enrichment of target genes of differentially expressed miRNAs was tested in the KEGG pathways through the DAVID software. The KEGG terms with corrected *p*-values less than 0.05 were significantly enriched terms, with the list of background genes of all porcine genes.

### 2.9. Interaction Networks of miRNAs and the Target Genes

Based on the String database (http://string-db.org/, accessed on 30 May 2022), we presented the interaction networks of the miRNAs and their target genes in the PPAR signaling pathway, and illustrated the relationships of the miRNAs with their target genes in this pathway. In addition, to demonstrate the relationships between differentially expressed miRNAs with their target genes, we used Cytoscape software to assess the relationships between miRNAs, the target genes that were significantly enriched in the GO and KEGG analyses and the target genes that were reported to be related to lipid metabolism in previous studies.

### 2.10. Transcription Factor Binding Sites (TFBS) Prediction of the Differentially Expressed miRNAs

The cis-acting elements that regulate gene expression are distributed 300–3000 bp upstream of the coding region. Based on the method described by Liu et al. [29], we selected 2000 bp upstream of ssc-miR-486, ssc-miR-133a-3p and ssc-miR-1 as the promoter sequences, and downloaded them from Ensembl (http://uswest.ensembl.org/, accessed on 30 May 2022) and the NCBI Database (http://www.ncbi.nlm.nih.gov/http://uswest.ensembl.org/, accessed on 30 May 2022). Then, these sequences were submitted to JASPAR CORE Vertebrate (http://jaspardev.genereg.net, accessed on 30 May 2022) and Scan Version 1.7 (http://www-bimas.cit.nih.gov/molbio/proscan/, accessed on 30 May 2022) for in silico analysis.

### 2.11. qRT-PCR Identification

The expression of the miRNAs was validated through the qRT-PCR method. With a SG One-Step miRNA RT Kit (#Q1014, SinoGene, Beijing, China), 2 µg of total RNA was reverse transcribed to cDNA. Then, SYBR Green real-time PCR was carried out using the synthesized cDNA as the template. The qRT-PCR reaction system comprised 7.5 µL of 2 × SG Green qRT-PCR Mix, 0.25 µL Forward Primer, 0.25 µL of Reverse Primer, 1 µL cDNA and 6 µL nuclease-free water. Then, the qRT-PCR reactions were performed by Step One PLUS (Applied Biosystems, Foster city, CA, USA) at 95 °C for 10 min, followed by 45 cycles of 95 °C for 15 s, 60 °C for 15 s, and, finally, dissociation analysis. The porcine U6 gene was used as an internal control, and the ΔΔCt method was used to calculate the relative expression level of the miRNAs between the samples. The qRT-PCR results showed a S-shaped amplification curve and a single-peak dissolution curve. This indicates that specific amplifications were achieved with the primers designed by Sinogene Scientific (Beijing, China).

### 2.12. Statistical Analysis

All data are shown as the means ± SD. A Student’s *t*-test was performed for comparison. A corrected *p* < 0.05 was considered statistically significant.

## 3. Results

### 3.1. miRNAs Sequencing and Mapping

The miRNA sequencing was performed on an Illumina Hiseq 2500 platform (the package contains HCS v2.0.12, RTA v1.17.21.3, Recipe Fragments v1.3.61, SAV 1.8.20 and BaseSpace Broker 2.0.13022.1628), generating reads from the L library and D library. A total of approximately 26,486,906 raw reads were obtained. There were 16,813,692 uniquely mapped reads after removing the low-quality reads, adaptors and all possible contaminations. Among them, 8,259,953 (64.09%) were from the L library and 8,553,739 (62.89%) were from the D library. The clean reads were mapped to the Rfam database and distributed among several main categories, including miRNAs, rRNAs, snRNAs, tRNAs, other small RNAs and unannotated small RNAs (Table 1). The highest proportion of reads mapped to unannotated small RNAs. The reads mapped to the miRNAs were the second most common (12.43% in the L library and 14.28% in the D library).

### 3.2. Identification of Known miRNAs and Novel miRNAs

After mapping to the Rfam database, we annotated conserved and novel miRNAs with the remaining sequence. In the Laiwu pigs, we identified 2660 known miRNAs and 584 novel miRNAs. In the Large White pigs, we identified 2634 known miRNAs and 561 novel miRNAs. Among them, 1851 miRNAs were common in both breeds. The novel miRNAs in this study were named by the following rule: species name-m (four-digit)-5p/3p, for example, ssc-m0644-3p. In the statistical analysis of the sequence length of the mature miRNAs, lengths were distributed at 22 nt. This was the typical size for Dicer-derived products, and was in line with the principle of miRNA processing.

### 3.3. Identification of Differentially Expressed miRNAs

The Laiwu pigs and the Large White pigs showed distinct fat depositions. To investigate the miRNAs responsible for the significant differences in the fat characteristics, we identified the differentially expressed miRNAs between the Laiwu pigs and the Large White pigs. The strict screening criteria are mentioned in the methods section. The results indicated that 17 known miRNAs, including ssc-miR-133a-3p, ssc-miR-486, ssc-miR-1 and ssc-miR-204, were up-regulated in the Laiwu pigs compared with the Large White pigs (Table S1). In addition, 22 known miRNAs, including ssc-let-7i and ssc-miR-27b-3p, were down-regulated in the Laiwu pigs compared with the Large White pigs (Table S2). Therefore, ssc-miR-133a-3p, ssc-miR-486, ssc-miR-1, ssc-let-7i and ssc-miR-27b-3 were abundantly expressed in the Laiwu pigs compared with the Large White pigs. We identified 55 differentially expressed novel miRNAs between the Laiwu pigs and the Large White pigs. These differentially expressed miRNAs were used for further prediction of the targets.

### 3.4. Target Gene Prediction of the Differentially Expressed miRNAs

The identification of miRNA targets can show the functions of the differentially expressed miRNAs identified in this study. A total of 36,259 genes were predicted as potential targets of the 95 differentially expressed miRNAs in the Laiwu and Large White pigs (Tables S3 and S4). miRNAs can bind partially complementary sequences on target mRNAs and result in the post-transcriptional regulation of gene expression. This can change the biological processes in which their target genes are involved. Therefore, in order to further explore the function of the differentially expressed miRNAs, we mainly utilized the targeted genes for the subsequent functional analysis.

### 3.5. Gene Ontology (GO) and KEGG Pathway Analysis of the Targeted Genes of Differentially Expressed miRNAs

**Figure 1:**
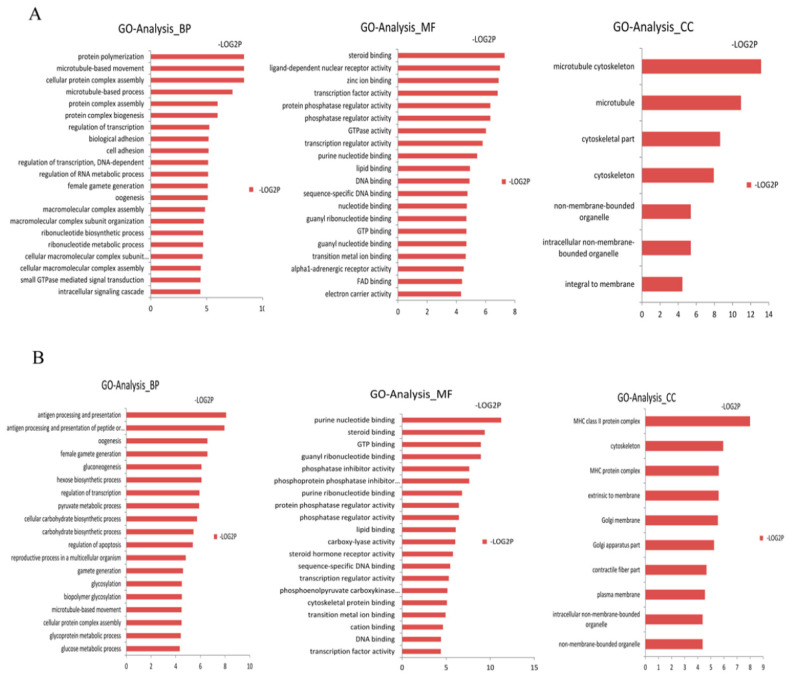
GO analysis of the targeted genes of the differentially expressed miRNAs. (**A**) represents the GO analysis of the targeted genes of the up-regulated differentially expressed miRNAs. (**B**) represents the GO analysis of the targeted genes of the down-regulated differentially expressed miRNAs. This figure is composed of 3 parts: biological processes (BP), molecular functions (MF) and cellular components (CC). The significance level of enrichment was *p* < 0.05.

We assigned GO terms to the targeted genes of differentially expressed miRNAs based on the sequence similarities of known proteins in the UniProt database, annotated with GO terms as well as the InterPro and Pfam domains they contain. We corrected the gene length bias through GO annotation and enrichment analysis on these targeted genes using the DAVID software (Tables S5 and S6). The GO terms (corrected *p*-value less than 0.05) were significantly enriched by the targeted genes. In Figure 1, the distribution of the biological processes, cellular components and molecular functions are shown. In the biological processes, the targeted genes of the up-regulated differentially expressed miRNAs in the Laiwu breed were significantly enriched in cellular protein complex assembly (GO: 0043623) and protein complex biogenesis (GO: 007027). The targeted genes of the down-regulated differentially expressed miRNAs in the Laiwu breed were significantly enriched in gluconeogenesis (GO: 000609), hexose biosynthetic processes (GO: 0019319), the carbohydrate biosynthetic process (GO: 0034637) and other GO terms related to material synthesis and energy metabolism. From the perspective of biological processes, the targeted genes of the up-regulated differentially expressed miRNAs were significantly enriched in cellular protein complex assembly (GO: 0043623) and protein complex biogenesis (GO: 007027). From the molecular function perspective, the targeted genes of the up-regulated and down-regulated differentially expressed miRNAs were mainly enriched in steroid binding (GO: 0005496), lipid binding (GO: 0008289) and protein phosphatase activity, which are related to the functions of enzyme activity and lipid binding. From the cellular component perspective, the cytoskeleton (GO: 0005856) and organelle (GO: 0043228) were significantly enriched by the targeted genes of both the up-regulated and down-regulated differentially expressed miRNAs.

**Figure 2:**
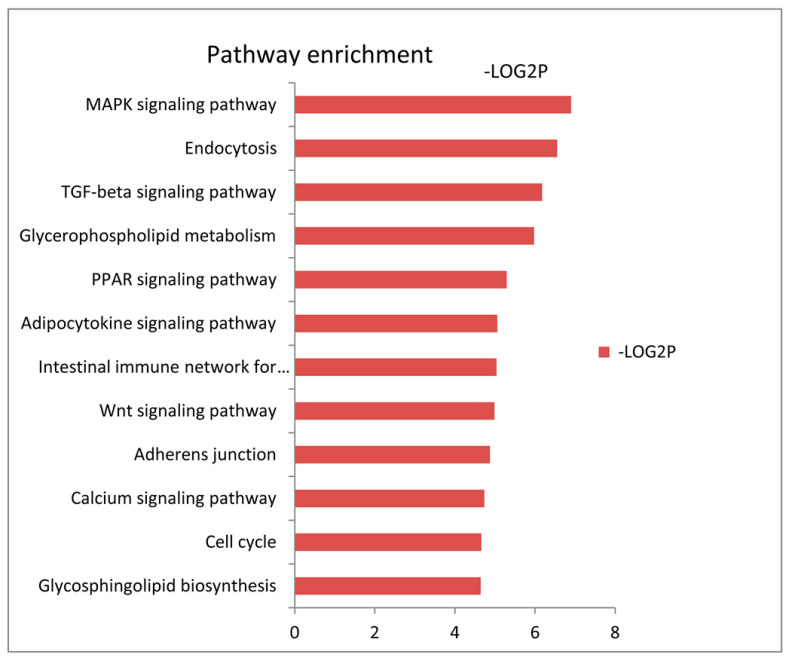
KEGG enrichment analysis of the targeted genes of the differentially expressed miRNAs. The horizontal axis refers to the –log2P, while the vertical axis refers to the significantly enriched pathway with a corrected *p* < 0.05. A higher number for –log2P value indicates the promoted performance of the pathway enrichment.

To probe the potential function of the miRNAs with differential expression, we tested the statistical enrichment of the targeted genes of the differentially expressed miRNAs in the Kyoto Encyclopedia of Genes and Genomes (KEGG) pathway, using DAVID software (Figure 2 and Table S7). In Figure 2, pathway enrichment indicates that the MAPK, PPAR and Wnt signaling pathways were the significantly enriched terms. This suggests that by targeting the genes in these pathways that are known to be involved in fatty acid metabolism, the differentially expressed miRNAs might regulate adipogenic differentiation and lipid metabolism.

### 3.6. Construction of the miRNA Target Network

**Figure 3:**
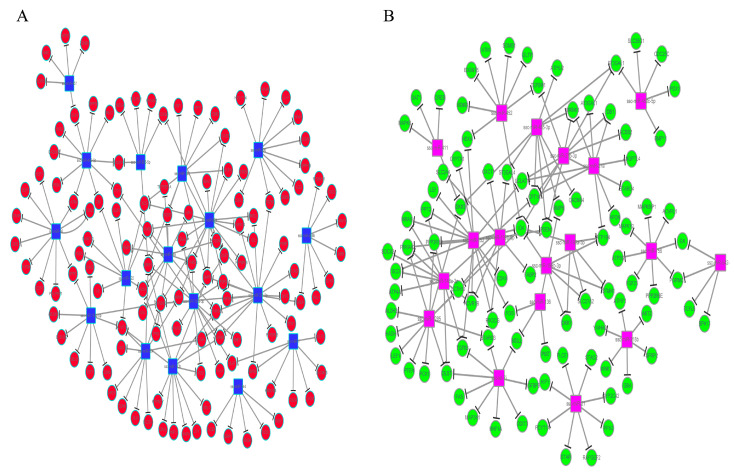
Relationships between the differentially expressed miRNAs and their targeted genes. (**A**) represents the relationships between the up-regulated miRNAs with their targeted genes. (**B**) represents the relationships between the down-regulated miRNAs with their targeted genes. The circular nodes are the targeted genes, while the square nodes represent the differentially expressed miRNAs.

To investigate the interaction between the miRNAs and their target genes, we selected the target genes enriched in significant GO BP terms and KEGG pathways, as well as genes related to fat deposition, to construct the miRNA target networks [30,31,32]. In Figure 3 and Table S8, one gene can be targeted by multiple miRNAs, and most miRNAs target more than one gene. For example, ssc-miR-455-3p was directed to the targeted genes Carnitine Palmitoyltransferase 1A (CPT1A) and DKK3, and ssc-miR-874 was directed to STARD3 and MGLL. Importantly, among these targeted genes, Stearoyl-CoA Desaturase (SCD), Stearoyl-CoA Desaturase 5 (SCD5), STARD3 and CPT1A were involved in adipocyte differentiation and fat deposition [30,31,32].

### 3.7. The Relationship between Differentially Expressed miRNAs and the Targeted Genes in the PPAR Pathway

**Figure 4:**
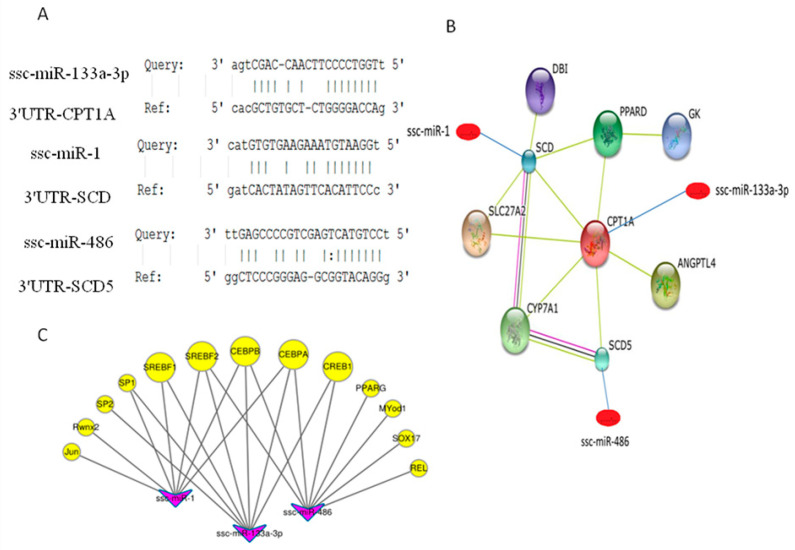
Relationships between ssc-miR-133a-3p, ssc-miR-1, ssc-miR-486 and the targeted genes CPT1A, SCD and SCD5, as well as the transcription factors binding to these miRNAs. (**A**) represents the result of the target gene prediction of ssc-miR-133a-3p, ssc-miR-1 and ssc-miR-486, using miRanda software. (**B**) represents the protein-protein interactions and the interactions of the miRNAs with the CPT1A, SCD and SCD5 genes in the PPAR pathway. (**C**) represents the interactions of ssc-miR-133a-3p, ssc-miR-1 and ssc-miR-486, with the transcription factors binding to them. The yellow nodes indicate the transcription factors, and the pink nodes indicate the miRNAs.

Three important factors in the PPAR signaling pathway, SCD, SCD5 and CPT1A, participate in adipocyte differentiation and fat deposition. They were predicted to be the targets of ssc-miR-1, ssc-miR-486 and ssc-miR-133a-3p, respectively (Figure 4A). In addition, the expressions of these three miRNAs were highly abundant. Therefore, we studied the relationships between these three differentially expressed miRNAs and the targeted genes in the PPAR pathway based on the STRING database. The complicated relationships between glycerol kinase (GK), peroxisome proliferator-activated receptor delta (PPARD), diazepam binding inhibitor (DBI), acyl-CoA binding protein, stearoyl-CoA desaturase (SCD), SCD5, cytochrome P450 family 7 subfamily A, polypeptide 1(CYP7A1), solute carrier family 27 member 2 (SLC27A2) and CPT1A in the PPAR pathway are shown in Figure 4B.

### 3.8. Transcription Factor Binding Sites (TFBS) Prediction

The predicted results indicated that the promoter regions of the precursors of ssc-miR-486, ssc-miR-133a-3p and ssc-miR-1 (Table 2) had multiple transcription factor binding sites, including Jun proto-oncogene (JUN), transcription factor AP-2 (AP-2), Sp1 transcription factor (SP1), Sp2 transcription factor (SP2), sterol regulatory element binding transcription factor 1 (SREBF1,SREBP1), sterol regulatory element binding transcription factor 2 (SREBF2,SREBP2), CCAAT/enhancer binding protein α (CEBPA), CCAAT/enhancer binding protein β (CEBPB), myogenic differentiation 1 (Myod1), SRY-box 17 (Sox17) and runt-related transcription factor 2 (RunX2) (Figure 4C). Among these transcription factors, SP1, SREBP1, SREBP2, CEBPA and CEBPB were involved in the proliferation and differentiation of preadipocytes and in lipid metabolism. These data suggest that these transcription factors might bind to the promoter regions of ssc-miR-486, ssc-miR-133a-3p and ssc-miR-1 to further regulate the adipose-related biological processes.

### 3.9. Validation of the Sequencing Data by qRT-PCR

Six differentially expressed miRNAs were randomly selected and further examined by qRT-PCR to validate the sequencing data (Table S9). The result showed that ssc-miR-486, ssc-miR-133a-3p, ssc-miR-122, ssc-miR-1, ssc-miR-204 and ssc-m0092-3p were significantly higher. These data are consistent with the sequencing results.

## 4. Discussion

More than a third of the world’s population now struggles with being overweight or obese [33]. Obesity is a well-known risk factor for metabolic diseases, such as T2DM, dyslipidemia, coronary heart disease, hypertension, non-alcoholic fatty liver disease (NAFLD) and stroke. It is also linked to dementia, obstructive sleep apnea and numerous types of cancers [34]. Therefore, studying the mechanism of fat deposition and adipocyte differentiation is of great significance for the treatment of obesity. Previous studies have focused on the mechanism of fatty deposits using rodents. For example, the rat 3T3-L1 cell line is usually used to study the differentiation of fat cells [35,36]. Moreover, obese mice are also considered an animal model to study fat metabolism diseases [37]. However, there is a gap in the fatty deposit locations and their formation between humans and rodents. Pigs have the maximum ability for fat deposition. They deposit fat in a similar way to humans. Therefore, pigs are an ideal animal model to study human diseases related to adipose tissue [13]. Recently, researchers have studied the mechanism of obesity using pigs, and made some progress [38]. Laiwu pigs and Large White pigs, as they have significant differences in adipopexis, were chosen as the animal models. The profiles of the miRNAs in the adipose tissue of these two different breeds were generated using RNA-seq to further study the potential molecular mechanism of adipopexis. The results showed that 40 miRNAs were up-regulated and 54 miRNAs were down-regulated in the Laiwu pigs. The bioinformatics analysis of these differentially expressed miRNAs provided more information on the identification, classification and possible targeted genes related to adipopexis.

Existing studies have shown that some differentially expressed miRNAs (down-regulated miRNAs: ssc-let-7i, ssc-miR-21 and ssc-miR-27b-3p; up-regulated miRNAs: ssc-miR-1, ssc-miR-133b, ssc-miR-133a-3p and ssc-miR-486) were responsible for regulating subcutaneous adipogenic differentiation [39,40,41]. ssc-miR-1 and ssc-miR-133a were widely present in skeletal muscle tissues, while ssc-miR-21, ssc-miR-27b-3p and the Let-7 family were shown to play important roles in adipose muscle and myocardial tissue. Evidence indicated that these miRNAs can be found in muscular tissues [42,43]. However, ssc-miR-1 and ssc-miR-133a, with highly abundant expression, were up-regulated in the adipose tissue of Laiwu pigs. In addition, ssc-miR-1 and ssc-miR-133a are related to adipocyte differentiation in humans and rats [44,45]. Therefore, ssc-miR-1 and ssc-miR-133a can increase fat metabolism in pigs. In addition, the concentration of miR-486 in the serum is correlated with T2DM and obesity. Therefore, miR-486, a circulating miRNA, may be related to metabolic disorders [46]. Thus, the up-regulation of ssc-miR-486 in Laiwu pigs may be a potential biomarker of obesity. In Figure 4B, ssc-miR-133a-3p, ssc-miR-1 and ssc-miR-486 can change the signal transduction in the PPAR signaling pathway by targeting the important genes CPT1A, SCD and SCD5, respectively. Therefore, they are involved in the regulation of biological processes related to adipogenesis.

Some differentially expressed miRNAs with low expression were associated with adipopexis, including ssc-miR-758, ssc-miR-144, ssc-miR-204 and ssc-miR-122. ssc-miR-122 was important in regulating cholesterol and maintaining the steady state of fatty acids. The depressed expression of miR-122 effectively reduced the cholesterol content in the serum [47]. Furthermore, the targeted control of ssc-miR-144 and ssc-miR-758 to the ATP-binding cassette sub-family A member 1 (ABCA1) gene resulted in an indirect regulation of the metabolism of cholesterol [48,49]. ssc-miR-204 inhibited the Runx2 gene, thereby accelerating the adipogenic differentiation of MPCs and BMSCs [50], as well as the up-regulation of ssc-miR-204. This indicates that it may activate adipocyte differentiation in pigs. In addition, ssc-miR-206, ssc-miR-331-3p, ssc-miR-451, ssc-miR-499-5p, ssc-miR-885 and ssc-miR-95 were up-regulated in Laiwu compared with Large White pigs. ssc-miR-218b, ssc-miR-411, ssc-miR-450c-5p, ssc-miR-452, ssc-miR-455-3p, ssc-miR-542-3p, ssc-miR-136 and ssc-miR-1285 were down-regulated in the subcutaneous tissue of Laiwu compared with Large White pigs. These miRNAs have been well studied in cancer, but rarely in lipometabolism [51,52,53,54].

In the GO enrichment analysis, the targeted genes of the differentially expressed miRNAs were enriched in carbohydrate biosynthesis, lipid biosynthesis, steroid binding, enzyme activity and other functions involved in lipometabolism and energy metabolism. The results of KEGG enrichment analysis indicated that the PPAR, MAPK, Wnt and TGF-β signaling pathways were enriched. Previous studies have shown that these signaling pathways are related to adipocyte differentiation and fat metabolism. MAPK signaling involves four pathways, including ERK1/2, JNK, p38 MAPK and ERK5/BMK1, which have played an important role in adipopexis [55,56,57,58]. The Wnt signaling pathway is involved in the fate determination of the fat cell, as well as adipogenic differentiation and lipometabolism [59,60]. Kennsell et al. argued that the Wnt signaling pathway can inhibit adipogenic differentiation, either by depending on β-catenin or not [61]. The TGF-β signaling pathway is also closely related with lipometabolism. Kim et al. found that miR-21 targeted the gene transforming growth factor β receptor 2 (TGFBR2) to regulate adipogenic differentiation in human stem cells [39]. In addition, we found that the PPAR signaling pathway is important in fatty acid metabolism, sterol metabolism and adipogenic differentiation. In Figure 4, CPT1A, SCD5 and SCD (SCD1) were enriched in this pathway, and they were targeted by ssc-miR-133a-3p, ssc-miR-486 and ssc-miR-1, respectively. SCD, with highly conserved sequences, is present in two forms of isomers, SCD1 and SCD5, in most vertebrates [62]. SCD1 is the limiting enzyme in the transformation of saturated fatty acids to monounsaturated fatty acids, and plays an important role in fatty acid biosynthesis [14,63]. SCD5 affects the components of aliphatic acid in the milk of Holstein cows, and it is an important regulatory gene in lipometabolism [64]. Carnitine palmitoyltransferase 1 (CPT1) has three different hypotypes, including CPT-1A, CPT-1B and CPT-1C. CPT1 is the limiting enzyme in the β-oxidation of free fatty acids. CPT1 catalyzes the long-chain fatty acyl coenzyme A and carnitine into acyl carnitine, which facilitates the oxidation of fatty acids in the mitochondria matrix [32]. Malandrino et al. found that CPT1A promotes the oxidation of fatty acids in fat cells and macrophages, thus reducing lipid accumulation and inflammation [65]. Therefore, CPT1A plays a role in the inhibition of fat deposition. Based on the evidence mentioned above, ssc-miR-133a-3p, ssc-miR-486 and ssc-miR-1 regulate lipid metabolism by targeting the genes CPT1A, SCD5 and SCD, respectively. Among them, ssc-miR-133a-3p can promote fat deposition in pigs by targeting the gene CPT1A. This is in line with the high expression and up-regulation of ssc-miR-133a-3p in Laiwu pigs. However, it is necessary to study ssc-miR-486 and ssc-miR-1 effect lipid metabolism and deposition in pigs by targeting SCD5 and SCD. In Table S9, six differentially expressed miRNAs were selected, and the expression of these RNAs was determined by RT-PCR to validate the RNA-seq data. Three animals of each of the two breeds were validated for the miRNA. The expression/fold change of each miRNA was compared between the Large White and Laiwu pig groups. The expression levels obtained by RT-PCR were consistent with the RNA-seq results. The results indicated that the identified miRNA-targeted genes can regulate the genes and pathways that are involved in adipogenesis. This can define the fat deposition differences between the two groups of pigs.

It has been shown that targeting PPARs is a potential way to treat NAFLDs (Fatty Liver Diseases, Susceptibility To) [66,67]. This study found that some miRNAs can regulate the PPAR pathway. This study helps us to understand the players involved in regulating this pathway, and can provide insights into better treatments for NAFLDs.

## 5. Conclusions

This paper studied the differentially expressed miRNAs in fat deposition in pigs. Thirty-nine known miRNAs and fifty-five novel miRNAs were identified. In the Gene Ontology and KEGG analyses, the target genes of the differentially expressed miRNAs were involved in the PPAR, MAPK and Wnt signaling pathways, and were related to lipometabolism. In addition, we first reported that ssc-miR-133a-3p, ssc-miR-486 and ssc-miR-1 regulate the target genes CPT1A, SCD5 and SCD, respectively, in the PPAR signaling pathway so that they can have an impact on lipidosis. These three miRNAs were upregulated in the Laiwu pigs compared to the Large White pigs. Regulation of the target genes CPT1A, SCD5 and SCD by ssc-miR-133a-3p, ssc-miR-486 and ssc-miR-1, respectively, may contribute to the molecular mechanism by which Laiwu pigs accumulate more fat when compared to Large White pigs. Of course, these three targets, as well as the mechanism by which these miRNAs regulate fat deposition, still need to be verified in future experiments. Our findings, both computational and experimental, provide valuable information regarding the miRNAs involved in fat deposition, and also reveal the molecular mechanism of gene regulation governing the development and physiology of adipose tissue in pigs. Moreover, our study provides data that might provide insight into obesity-related diseases.

## Figures and Tables

**Table 1:** Statistics of the classification of the small RNAs.

Type	D Libraries	L Libraries
Unannotation	6,463,227	6,054,417
miRNA	1,221,683	1,026,545
rRNA	229,610	234,485
snRNA	189,944	156,546
tRNA	222,328	564,823
other	226,947	223,137

**Table 2:** The precursor information of ssc-miR-486, ssc-miR-133a-3p and ssc-miR-1.

Name	Accession	Chromosome	Location	Length (bp)
ssc-mir-486-1	NC_010459	17	12191266–12191345	80
ssc-mir-486-2	ENSSSCG00000022807	17	12191229–12191310	82
ssc-mir-133a-1	ENSSSCG00000018790	17	69274653–69274755	103
ssc-mir-133a-2	ENSSSCG00000026111	6	99485202–99485288	87
ssc-mir-1	ENSSSCG00000019004	17	69285393–69285500	108

## Data Availability

All the data are presented in the main manuscript and are available to readers.

## Supplementary Materials

The following are available online at https://www.mdpi.com/article/10.3390/genes14010094/s1, Table S1. Up-regulated expressed miRNAs between the Laiwu pig and Large white adipose tissue; Table S2. Down-regulated expressed miRNAs between the Laiwu pig and Large white adipose tissue; Table S3. Target genes of up-regulation expressed miRNAs between the Laiwu pig and Large white adipose tissue; Table S4. The target genes of down-regulation expressed miRNAs between the Laiwu pig and Large white adipose tissue; Table S5. GO analyses of the target genes of up-regulation expressed miRNAs in Laiwu pig and Large white adipose tissue; Table S6. GO analyses of the target genes of down-regulation expressed miRNAs in Laiwu pig and Large white adipose tissue; Table S7. KEGG enrichment analyses of the target genes of differentially expressed miRNAs in Laiwu pig and Large white adipose tissue; Table S8 The genes regulated by various miRNAs and the differential expression status of the miRNAs; Table S9. Validation of sequencing data by qRT-PCR.
