# Supplementary material for: Integrative Analysis of miRNAs Involved in Fat Deposition in Different Pig Breeds
Source: Genes (Basel). 2022 Dec 28;14(1):94. doi: 10.3390/genes14010094 (PMC9859024; doi:10.3390/genes14010094)
Supplement: Supplementary file 1 [file genes-14-00094-s001.zip › Table S8 The genes regulated by various miRNAs.pdf]

Table S9: The genes regulated by various miRNAs and the differential expression status of the miRNAs.

Gene name	Gene	Targeting miRNA	Up/Downregulated
carnitine palmitoyltransferase 1A	CPT1A	ssc-miR-455-3p	Down
		ssc-miR-27b-3p	Down
		ssc-miR-133a-3p	Up
StAR-related lipid transfer domain protein 3	STARD3	ssc-miR-1285	Down
		ssc-miR-874	Up
		ssc-let-7i	Down
Hexosaminidase A	HEXA	ssc-miR-122	Up
		ssc-miR-486	Up
Dickkopf-related protein 3	DKK3	ssc-miR-1285	Down
		ssc-miR-455-3p	Down
		ssc-miR-411	Down
Monoacylglycerol lipas	MGLL	ssc-miR-331-3p	Up
		ssc-miR-874	Up
		ssc-let-7i	Down
stearoyl-CoA desaturase	SCD	ssc-miR-1	Up
		ssc-miR-122	Up
stearoyl-CoA desaturase 5	SCD5	ssc-miR-486	Up
		ssc-miR-128	Up
